# Clinical aspects for differential diagnosis of Kawasaki disease shock syndrome: a case control study

**DOI:** 10.1186/s12887-020-02488-w

**Published:** 2021-01-08

**Authors:** Woo Young Park, Sang Yun Lee, Gi Beom Kim, Mi Kyoung Song, Hye Won Kwon, Eun Jung Bae, Eun hwa Choi, June Dong Park

**Affiliations:** Department of Pediatrics, Seoul National University Children’s Hospital, 101, Daehak-ro, Jongno-gu, Seoul, Republic of Korea

**Keywords:** Kawasaki disease shock syndrome, Kawasaki disease, Septic shock, Toxic shock syndrome

## Abstract

**Background:**

Because of the absence of a specific diagnostic test and pathognomonic clinical features, physicians must rely on the presence of specific clinical criteria and laboratory data that support the diagnosis of KD. To help clinicians distinguish KD, KDSS, septic shock, and TSS earlier, we suggest differential diagnosis and treatment guideline.

**Methods:**

Medical records of immunocompetent patients who were admitted to the pediatric department with a diagnosis of KDSS, septic shock or TSS (SS group) were retrospectively reviewed. In addition, KD patients were selected by seasonal matching to each case of KDSS patient by date of admission (± 2 weeks).

**Results:**

There were 13 patients with KDSS, 35 patients with SS group, and 91 patients with KD. In comparison between KDSS and septic shock group, KDSS group had significantly higher rate of coronary aneurysm incidence, and higher left ventricle dysfunction rate. In comparison between KDSS and TSS, patients with KDSS had a significantly higher erythrocyte sedimentation rate (ESR) and significantly lower creatinine. Receiver operation characteristic curve revealed that the optimal ESR cut off value for determining the KDSS was 56.0 (sensitivity 75.0%, specificity of 100.0%) and the optimal creatinine cut off value for determining the TSS was 0.695 (sensitivity 76.9%, specificity 84.6%).

**Conclusions:**

Clinical symptoms, laboratory finding, echocardiography, and culture studies can be used to differentiate KD, KDSS, septic shock and TSS.

**Supplementary Information:**

The online version contains supplementary material available at 10.1186/s12887-020-02488-w.

## Background

Kawasaki disease (KD) is an acute systemic inflammatory vasculitis of early childhood, predominantly involving medium-sized arteries [1]. About 15–25% of untreated children will develop coronary artery abnormalities [2]. Early detection and prompt initiation of therapy with high dose intravenous immunoglobulin (IVIG) plus aspirin can reduce the incidence of serious coronary artery complications. Therefore, accurate and timely diagnosis of KD is critical. However, because of the absence of a specific diagnostic test and pathognomonic clinical features, physicians must rely on the presence of specific clinical criteria and laboratory data that support the diagnosis of KD, while excluding other illnesses that could mimic the disease [3].

As the COVID-19 pandemic continues, there have been reports of multi-system inflammatory syndrome in children (MIS-C) that shares clinical features with KD [4]. Recognition of sudden increase of severe Kawasaki-like disease in the North America and European countries during the COVID-19 pandemic is alarming given the unknown etiology of KD. Also, it is timely to address the differences in clinical manifestations between various Kawasaki mimic diseases as some patients with KD may also present with hypotension or shock, known as “Kawasaki disease shock syndrome” (KDSS). Differentiating between KDSS and septic shock or toxic shock syndrome (TSS) in early stages of clinical diagnosis is challenging [1]. Although KDSS and septic shock or TSS show similar clinical presentation, early diagnosis is critical in patients with unstable conditions because different clinical management is required for treatment.

Therefore, we retrospectively investigated patients admitted with diagnosis of KDSS, septic shock or TSS (SS group), and KD among immunocompetent patients. Additionally, we tried to suggest KDSS differential diagnosis and treatment guideline.

## Methods

### Patient selection

A computerized search program was used to search for patients diagnosed with KD or KDSS or SS that occurred in immunocompetent patients from January 2004 to August 2019. These patients were classified into three groups: KDSS group, KD group, and SS group. 13 patients satisfied the KDSS criteria [5]. In KD group, control subjects were chosen for each case patient and matched to its control by date of admission (± 2 weeks) as the matching factor to control for the possibility of seasonal variation. Patients with incomplete KD and refractory KD were also included in the KD group. Ninety-one patients met the inclusion criteria [6]. To search for patients with septic shock group, we searched “Septic shock” and “Toxic shock syndrome” from discharge records and immunocompromised patients were excluded. A total of 29 patients met the inclusion criteria for the septic shock or toxic shock syndrome.

This study used clinical data retrieved from Seoul National University Hospital Patients Research Environment system. This study was approved by the Institutional Review Board of the Seoul National University Hospital (IRB number: 1910–051-1068, approved date: Oct 14th, 2019).

### Definitions

Diagnosis of KD was based on the diagnostic criteria provided by the American Heart Association (AHA) [7]. The diagnostic criteria were the presence of a fever lasting for at least five days and at least four of the following five typical clinical features of KD: bilateral bulbar conjunctival injection without exudate, erythema and cracking of lips, strawberry tongue, and/or erythema of oral and pharyngeal mucosa, polymorphous rash and cervical lymphadenopathy (≥ 1.5 cm in diameter), usually unilateral. A diagnosis of incomplete or atypical KD was used for patients with a history of fever lasting for more than five days who had less than four of the five typical clinical features of KD but showing evidence of coronary artery lesion on echocardiography or compatible laboratory findings suggested by AHA [7]. KDSS was defined as a patient with KD complicated by hypotension without evidence of infection [5]. Hypotension was defined as a systolic blood pressure < 5th percentile for a patient’s age. All patients with TSS were diagnosed by infection specialists using the diagnostic criteria provided by the Centers for Disease Control and Prevention (Supplement Table 1) [8]. Septic shock was defined as sepsis and cardiovascular organ dysfunction. Cardiovascular organ dysfunction means hypotension (< 5th percentile for age) despite > 40 ml/kg fluid bolus in 1 h or vasoactive requirement to maintain blood pressure despite > 40 ml/kg fluid bolus in 1 h or two or more signs of abnormal perfusion (increased lactate, metabolic acidosis, decreased urine output (< 0.5 mL/kg/hr), capillary refill > 5 s) [9]. Organ damage was defined as damage in major organs including brain, heart, lung, liver, and kidney due to hypoperfusion and sequential organ failure assessment score was used as an index [10]. The coronary artery aneurysm was defined as Z score ≥ 2.5. In the present study, patients with septic shock and TSS were assigned into the septic shock (SS) group.

### Data collection

For all patients with KD, we accessed the following retrospectively collected data: demographic data (age, gender, associated symptoms and number of days of fever at presentation), laboratory values (white blood cell and differential counts, platelet count, erythrocyte sedimentation rate (ESR), and concentrations of hemoglobin, C-reactive protein (CRP), aspartate aminotransferase (AST), alanine aminotransaminase (ALT), and γ-glutamyl transpeptidase (GGT), creatinine, blood urea nitrogen (BUN), albumin, electrolytes), laboratory evaluations of coagulation function, response to IVIG, oral prednisolone, methylprednisolone pulse therapy, and infliximab, and echocardiographic data. The laboratory data were compared to the worst values between 3 groups.

### Statistical analysis

All data analyses were performed using SPSS statistics 25.0 (IBM, Armonk, NY, USA). Data is presented as median and ranges if not normally distributed. Not normally distributed data was compared between groups using the Mann-Whitney test, Fisher’s exact test, or Chi-square test. One-way ANOVA was performed for continuous variables if normally distributed. Kruskal-Wallis test was used to compare the laboratory data among more than two groups. Area under the ROC (receiver operating characteristic) curve was used as an accuracy index for the diagnosis of KDSS. As appropriate, *p* < 0.05 was considered significant.

## Results

From January 2004 to August 2019, 13 patients were diagnosed as a KDSS. Over the same time, 13 patients were admitted with a diagnosis of TSS and 16 patients were admitted with a diagnosis of septic shock. In addition, 91 patients were treated as KD. Demographics and clinical characteristics for KDSS group and control groups are shown in supplement Table 2.

**Fig. 1:**
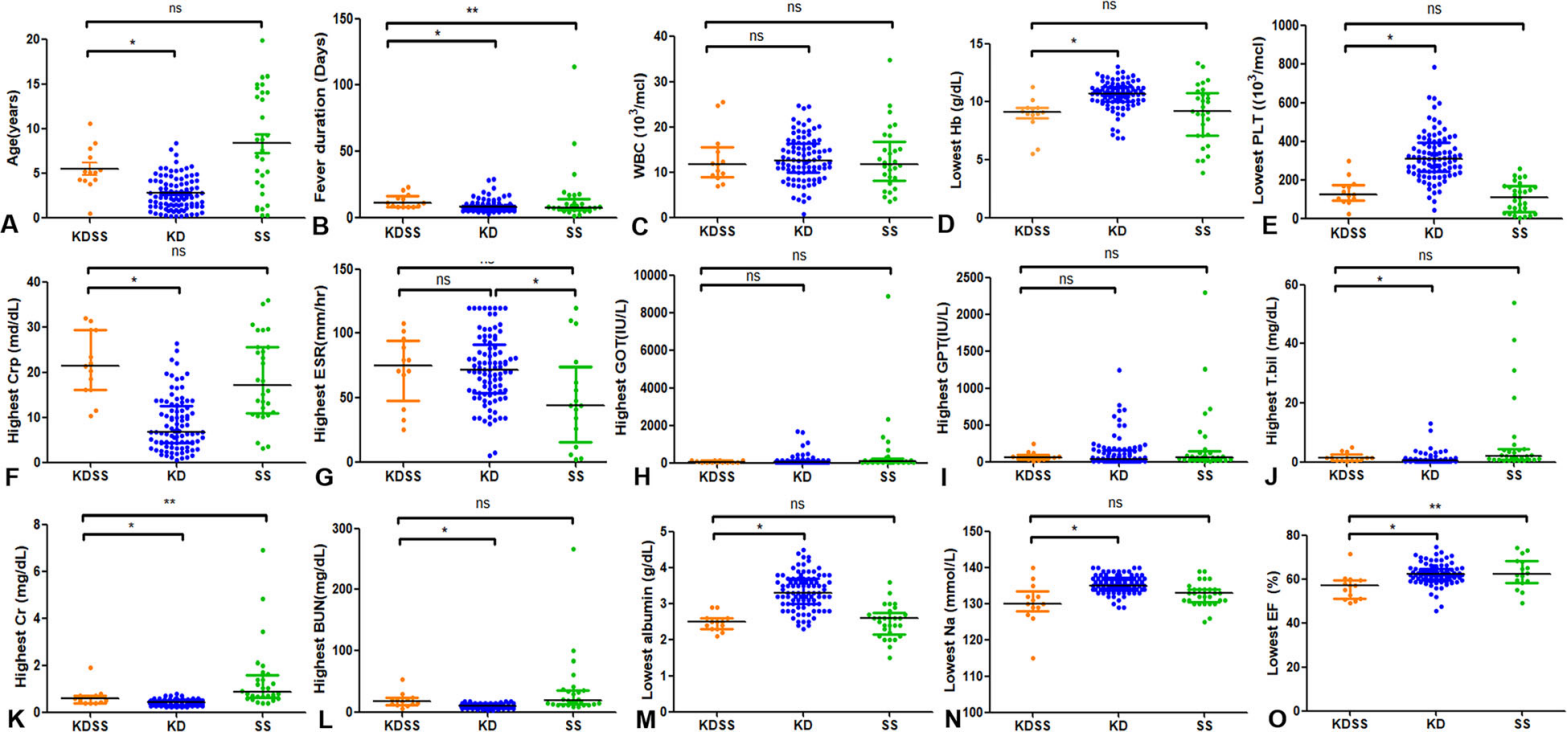
Comparison of Age, Fever duration, Laboratory Results and Ejection fraction in 3 Different Patient Groups. Statistics were calculated with GraphPad Prism software. Horizontal lines represent median values for each group and vertical lines represent interquartile range. *, **: statistically significant, (*p* < 0.05), as determined by the Kruskal-Wallis test. ns: not significant

All patients in the KDSS and KD group, and 17 of 29 patients in the SS group received an echocardiographic assessment. Laboratory values and ejection fraction for KDSS group and control groups are shown in Fig. 1 and supplement Table 3. Kawasaki features, associated symptoms and treatment for KDSS group and control groups are shown in supplement Table 4.

### KDSS vs. KD

Demographics and clinical characteristics of patients with Kawasaki disease shock syndrome and Kawasaki disease are shown in Table 1. Patients with KDSS had a significantly older age (*p* = 0.000), longer fever duration (*p* = 0.002), longer hospital day (*p* = 0.000), longer ICU admission days (*p* = 0.000), and higher rate of using inotropic drugs (*p* = 0.000) than patients with KD. However, there were no significant differences in sex-distribution between the two groups.

**Table 1:** Demographics and clinical characteristics of patients with Kawasaki disease shock syndrome and Kawasaki disease (continuous variables were described as median and range)

	KDSS (*n*=13)	KD (*n*=91)	*P* value
Age, (years)	5.1 (0.5–10.6)	2.3 (0.2–8.4)	0.000
Male (%)	46.2% (6/13)	58.2% (53/91)	0.411
Fever duration (days)	11 (8–23)	8 (3–29)	0.002
Total hospital day (days)	18 (6–31)	6 (3–18)	0.000
Follow up duration (months)	10 (1–105)	13 (0–175)	0.723
ICU care (%)	53.8% (7/13)	0.0% (0/91)	0.000
ICU care duration (days)	4 (0–11)	0	0.000
Inotropic drugs (%)	92.3% (12/13)^§^	1.1% (1/91)	0.000
Respiratory support			
- No support	30.8% (4/13)	100% (91/91)	0.000
- Oxygen delivery	53.8% (7/13)	0.0% (0/91)	0.000
- Mechanical ventilation	15.4% (2/13)	0.0% (0/91)	0.015
Mortality (%)	0.0% (0/13)	0.0% (0/91)	

*KDSS* Kawasaki disease shock syndrome, *KD* Kawasaki disease, *SS* Septic shock and toxic shock syndrome, *ICU* Intensive care unit

Kawasaki features and associated symptoms of Kawasaki disease shock syndrome and Kawasaki disease are shown in Table 2. There was no statistically significant difference in Kawasaki features between KDSS and KD groups. Patients in the KDSS group had significantly higher gastrointestinal symptoms (*p* = 0.000), respiratory symptoms (*p* = 0.000), systemic pain (*p* = 0.000), pleural effusion (*p* = 0.00), and organ damage (*p* = 0.000) than those in the KD group.

**Table 2:** Kawasaki features and associated symptoms of Kawasaki disease shock syndrome and Kawasaki disease

	KDSS(*n*=13)	KD(*n*=91)	*P* value
Kawasaki features			
Conjunctival injection	92.3% (12/13)	91.2% (83/91)	1.000
Oropharyngeal changes	84.6% (11/13)	81.3% (74/91)	1.000
Polymorphous rash	76.9% (10/13)	84.6% (77/91)	0.442
Cervical lymphadenopathy	69.2% (9/13)	44.0% (40/91)	0.088
Extremity changes	92.3% (12/13)	67.0% (61/91)	0.102
Associated symptoms			
Gastrointestinal symptoms	84.6% (11/13)	26.4% (24/91)	0.000
Respiratory symptoms	76.9% (10/13)	9.9% (9/91)	0.000
Neurologic symptoms	15.4% (2/13)	2.2% (2/91)	0.066
Systemic pain	53.8% (7/13)	3.3% (3/91)	0.000
Pleural effusion	76.9% (10/13)	2.2% (2/91)	0.000
Organ damage	92.3% (12/13)	20.9%(19/91)	0.000

*KD* Kawasaki disease, *KDSS* Kawasaki disease shock syndrome, *SS* Septic shock and toxic shock syndrome

Diagnosis and treatment of Kawasaki disease shock syndrome and Kawasaki disease are shown in Table 3. KD group had significantly higher rate of initial diagnosis of KD than those in the KDSS group and KDSS group initially showed only 1–2 of typical Kawasaki symptoms. However, 3 or more symptoms were seen in all patients eventually. In addition, patients in the KDSS group had significantly higher IVIG resistance rate than those in the KD group.

**Table 3:** Diagnosis, and treatment of Kawasaki disease shock syndrome and Kawasaki disease

	KDSS(*n*=13)	KD(*n*=91)	*P* value
Diagnosis			
Initial diagnosis of KD	23.1% (3/13)	80.2% (73/91)	0.000
Complete KD	46.2% (6/13)	67.0% (61/91)	0.214
Incomplete KD	38.5% (5/13)	29.7% (27/91)	0.532
Treatment			
1st IVIG	84.6% (11/13)	98.9% (90/91)	0.041
2nd IVIG	76.9% (10/13)	19.8% (18/91)	0.000
Oral prednisolone	7.7% (1/13)	11.0% (10/91)	1.000
Methylprednisolone pulse therapy	38.5% (5/13)	11.0% (10/91)	0.020
Infliximab	15.4% (2/13)	0.0% (0/91)	0.015
Antibiotics	92.3% (12/13)	15.4% (14/91)	0.000

*IVIG* Intravenous immunoglobulin, *KD* Kawasaki disease, *KDSS* Kawasaki disease shock syndrome, *SS* Septic shock and toxic shock syndrome

Compared to those in the KD group, patients in the KDSS group had lower hemoglobin level (*p* = 0.000), lower platelet counts (*p* = 0.00), higher C-reactive protein level (*p* = 0.000), higher total bilirubin level (*p* = 0.012), higher creatinine level (*p* = 0.001), higher BUN level (*p* = 0.000), lower albumin level (*p* = 0.00), and lower sodium level (*p* = 0.000) (Fig. 1, supplementary Table 3).

Compared with patients with hemodynamically normal KD, left ventricle dysfunction represented by reduced ejection fraction (< 55%) was more common in the KDSS group (*p* = 0.000). In addition, there was significantly (*p* = 0.040) more coronary artery dilatation in the KDSS group than in the KD group. In the KDSS group, during the acute phase, five patients showed transient coronary artery dilatation and three patients had persistent coronary artery dilatation. In the KD group, during the acute phase of Kawasaki disease, 19 patients showed transient coronary artery dilatation and four patients had persistent coronary artery dilatation at the last echocardiography.

### KDSS vs SS group

Demographics and clinical characteristics of patients with Kawasaki disease shock syndrome and septic shock group are shown in Table 4. There were no significant differences in sex-distribution, age, fever duration, total hospital day, ICU admission days, or rate of using inotropic drugs between the two groups.

**Table 4:** Demographics and clinical characteristics of patients with Kawasaki disease shock syndrome and septic shock group (continuous variables were described as median and range)

	KDSS (*n*=13)	SS (*n*=29)	*P* value
Age, (years)	5.1 (0.5–10.6)	7.6 (0.3–19.9)	0.185
Male (%)	46.2% (6/13)	65.5% (19/29)	0.237
Fever duration (days)	11 (8–23)	7 (1–114)	0.068
Total hospital day (days)	18 (6–31)	13 (7–225)	0.419
Follow up duration (months)	10 (1–105)	2.5 (0–119)	0.124
ICU care (%)	53.8% (7/13)	62.1% (18/29)	0.616
ICU care duration (days)	4 (0–11)	3 (0–20)	0.591
Inotropic drugs (%)	92.3% (12/13)^§^	86.2% (25/29)	0.153
Respiratory support			
- No support	30.8% (4/13)	48.3% (14/29)	0.289
- Oxygen delivery	53.8% (7/13)	10.3% (3/29)	0.005
- Mechanical ventilation	15.4% (2/13)	41.4%(12/29)	0.159
Mortality (%)	0.0% (0/13)	10.3%(3/29)	0.540

*KDSS* Kawasaki disease shock syndrome, *KD* Kawasaki disease, *SS* Septic shock and toxic shock syndrome, *ICU* Intensive care unit

Kawasaki features and associated symptoms of patients with Kawasaki disease shock syndrome and septic shock group are shown in Table 5. Patients with KDSS had significantly higher rate of conjunctival injection (*p* = 0.000), oropharyngeal changes (*p* = 0.000), cervical lymphadenopathy (*p* = 0.000), and extremity changes (*p* = 0.001) than patients with SS group. In addition, patients with KDSS had a significantly higher rate of respiratory symptoms (*p* = 0.033) and pleural effusion (*p* = 0.033) than patients with SS group.

**Table 5:** Kawasaki features and associated symptoms of Kawasaki disease shock syndrome, and Septic shock group

	KDSS(*n*=13)	SS (*n*=29)	*P* value
Kawasaki features			
Conjunctival injection	92.3% (12/13)	10.3% (3/29)	0.000
Oropharyngeal changes	84.6% (11/13)	17.2% (5/29)	0.000
Polymorphous rash	76.9% (10/13)	69.0% 1 (20/29)	0.722
Cervical lymphadenopathy	69.2% (9/13)	3.4%(1/29)	0.000
Extremity changes	92.3% (12/13)	34.5% (10/29)	0.001
Associated symptoms			
Gastrointestinal symptoms	84.6% (11/13)	82.8% (24/29)	1.000
Respiratory symptoms	76.9% (10/13)	41.4% (12/29)	0.033
Neurologic symptoms	23.1% (3/13)	51.7% (15/29)	0.083
Systemic pain	53.8% (7/13)	37.9% (11/29)	0.335
Pleural effusion	76.9% (10/13)	41.4% (12/29)	0.033
Organ damage	92.3% (12/13)	82.8% (24/29)	0.647

*KD* Kawasaki disease, *KDSS* Kawasaki disease shock syndrome, *SS* Septic shock and toxic shock syndrome

Diagnosis and treatment of Kawasaki disease shock syndrome and septic shock group are shown in Table 6. Patients with KDSS had significantly higher rates of usage 2nd IVIG (*p* = 0.000), and methylprednisolone pulse therapy (*p* = 0.002) than patients with SS group. There was no difference in antibiotic treatment rates between the two groups. 12 of the 13 patients in the KDSS were initially treated with antibiotics as they were indistinguishable from septic shock.

**Table 6:** Diagnosis, and treatment of Kawasaki disease shock syndrome, and Septic shock group

	KDSS(*n*=13)	SS (*n*=29)	*P* value
Diagnosis			
Initial diagnosis of KD	23.1% (3/13)	0%(0/29)	0.025
Complete KD	46.2% (6/13)	0%(0/29)	0.000
Incomplete KD	38.5% (5/13)	3.4%(1/29)	0.007
Treatment			
1st IVIG	84.6% (11/13)	51.7% (15/29)	0.084
2nd IVIG	76.9% (10/13)	17.2% (5/29)	0.000
Oral prednisolone	7.7% (1/13)	0.0%(0/29)	0.310
Methylprednisolone pulse therapy	38.5% (5/13)	0.0%(0/29)	0.002
Infliximab	15.4% (2/13)	0.0%(0/29)	0.091
Antibiotics	92.3% (12/13)	100.0%(29/29)	0.310

*IVIG* Intravenous immunoglobulin, *KD* Kawasaki disease, *KDSS* Kawasaki disease shock syndrome, *SS* Septic shock and toxic shock syndrome

Compared with KDSS patients, patients in septic shock group had higher level of creatinine (*p* = 0.006) and lower ESR level (*p*=0.008). However, there were no significant differences in hemoglobin, platelet count, CRP level, liver function, BUN, albumin or sodium level between KDSS and SS groups. In addition, there was no coronary artery aneurysm case in SS group. (Fig. 1, Supplement Table 3).

### KDSS vs TSS

**Fig. 2:**
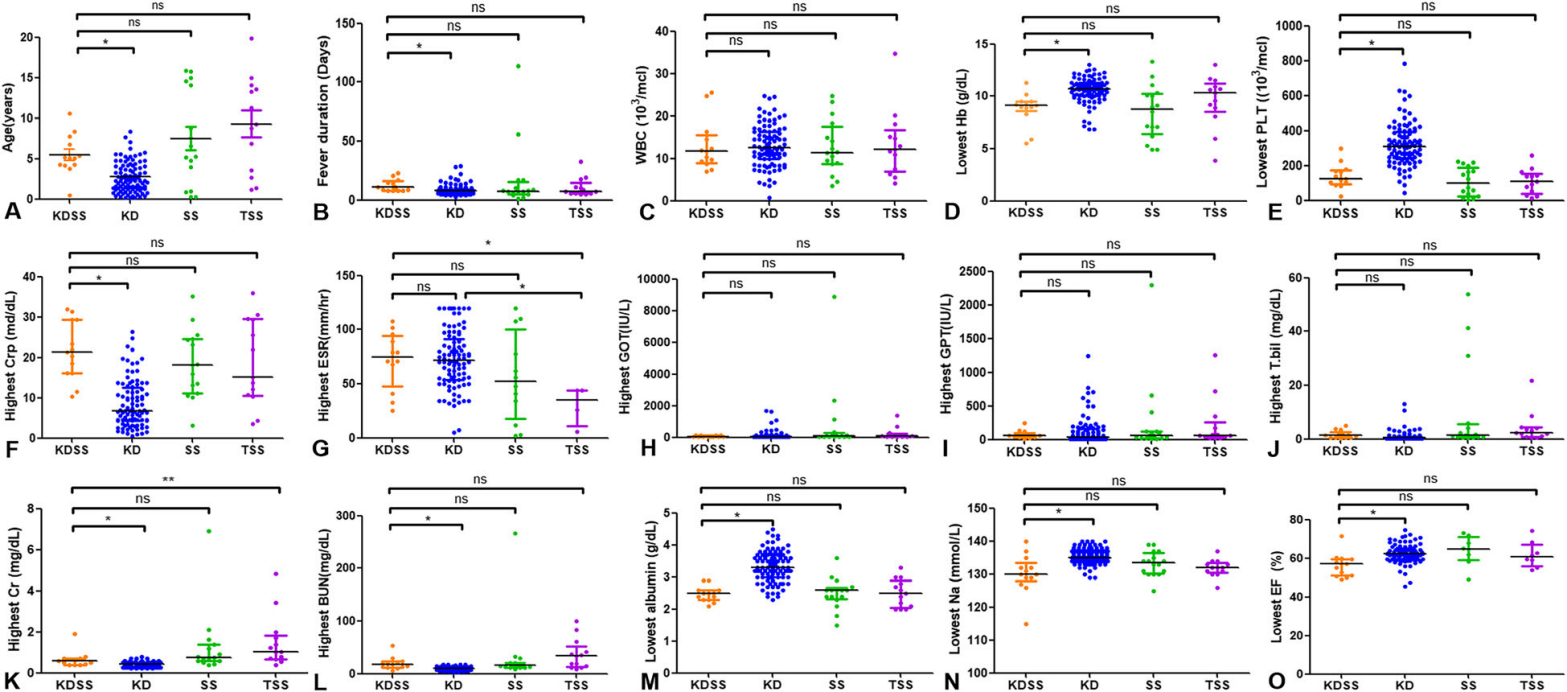
Comparison of Age, Fever duration, Laboratory Results and Ejection fraction in 4 Different Patient Groups. Statistics were calculated with GraphPad Prism software. Horizontal lines represent median values for each group and vertical lines represent interquartile range. *, **: statistically significant, (*p* < 0.05), as determined by the Kruskal-Wallis test. ns: not significant

**Fig. 3:**
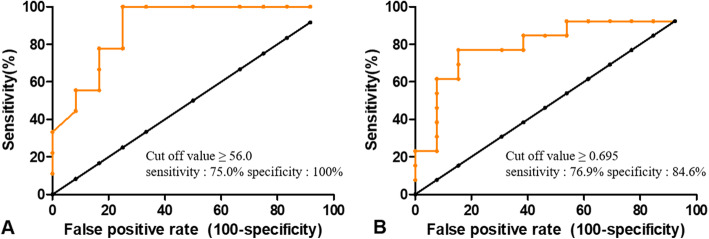
Receiver operation chaaracterisic (ROC) curve and cutoff value for determining disease. **a** The optimal ESR cutoff for determining the KDSS ≥ 56.0, sensitivity: 75.0% and specificity: 100.0%, AUC, 0.894; 95% CI, 0.757–1.000, *P*=0.003, **b** The optimal creatinine cutoff for determining the TSS ≥ 0.695, sensitivity: 76.9% and specificity: 84.6%, AUC, 0.802; 95% CI, 0.620–0.983, *P*=0.009

There were no significant differences in age, sex-distribution, and laboratory findings between KDSS and TSS groups except for ESR and creatinine. Patients with KDSS had a significantly higher ESR (*p* = 0.019) and significantly lower creatinine (*p*=0.007) than patients with TSS. (Supplement Table 3) Receiver operation characteristic (ROC) curve revealed that the optimal ESR cut off value for determining the KDSS was 56.0 which had a sensitivity of 75.0% and a specificity of 100.0% (Figs. 2 and 3a, AUC, 0.894; 95% CI, 0.757–1.000, *P*=0.003) and the optimal creatinine cut off value for determining the TSS was 0.695 which had as sensitivity of 76.9% and a specificity of 84.6% (Figs. 2 and 3b, AUC, 0.802; 95% CI, 0.620–0.983, *P*=0.009).

## Discussion

Because there is no pathognomonic clinical feature or diagnostic test for KD, patients with KDSS are frequently misdiagnosed. Its clinical presentation may be mistaken for septic shock or TSS, leading to delay in treatment [11]. In COVID-19 pandemic period, cases for MIS-C have been reported and shared clinical features with KD, KDSS, and TSS [4]. Case definitions for emerging inflammatory condition during COVID-19 pandemic from the World Health Organization, Royal College of Pediatrics and Child Health, and Centers for Disease Control and Prevention are similar in many ways to KD [12–14]. Therefore, it has become more important to accurately distinguish among various Kawasaki mimicking diseases.

Considering the symptoms of KDSS that look different from KD and similar symptoms to other diseases such as TSS or MIS-C, the pathophysiological cause becomes curious. Despite 4 decades of investigation, the cause of KD remains unknown [7]. Recently updated three major pathophysiologic components of KD are a genetic predisposition, immunomodulation through both habitual exposures and environmental factors, and contact with the disease trigger or triggers [15]. In this background, exposure to the still unidentified trigger such as SARS-CoV-2 might result in the development of KD in a genetically susceptible child, with at least a partial contribution from immune-modulating factors. Multiple factors may act sequentially or simultaneously as predisposing, immune-modulating, or triggering agents, altering both individual risk as well as the incidence of KD in the population across countries or regions [15]. Recent reports of MIS-C suggest that MIS-C may have a different racial predilection, affecting primarily people of African American, Caribbean, and Hispanic ancestry [4].

The pathophysiology of KDSS is unknown, but it is hypothesized that the “overexpression” of proinflammatory cytokines, in combination with an intense and systemic inflammation, might lead to multiple organ damage and failure in KDSS. These clinical and laboratory findings suggest greater underlying inflammation with a more intense systemic vasculitis, capillary leak, and more profound myocardial involvement [1, 5, 11, 16].

Gámez-González et al. [17] reported that patients with KDSS seem to have more gastrointestinal symptom, incomplete presentation, IVIG resistance and worse cardiac outcomes. These findings were also confirmed in our study. In addition, in our study, patients with KDSS seem to have more respiratory symptoms and systemic pain as well.

Because there is no diagnostic test for KD, the diagnosis is made based on patient’s symptoms. However, symptoms of KD do not appear at once and may appear in other diseases such as TSS and septic shock, making it difficult to diagnose. Thus, for many years, studies to distinguish KD from other diseases have been conducted. Zandstra, Judith, et al. [18] reported that combination of plasma markers, myeloid-related protein 8/14, CRP, and human neutrophil-derived elastase may assist in distinguishing KD from other infection. However, in our study, there was no significant difference in CRP between KDSS and SS group. These inflammatory markers are still insufficient to confirm KD and further studies are needed.

In actual clinical trials, it was difficult to distinguish between KDSS and TSS. Therefore, we studied the differential diagnosis between KDSS and TSS and performed analysis among subgroups. In comparison of clinical manifestations between KDSS and TSS, no patients with TSS showed conjunctival injection or cervical lymphadenopathy, which could be the points of differential diagnosis. In laboratory tests, highest ESR and creatinine level showed significant difference and possibility as useful marker for differential diagnosis in two groups. (Figs. 2 and 3) These results are novel findings, and different from previous studies [1]. Since the diagnostic criteria for TSS include elevated creatinine, it is not surprising that the TSS group has a significantly higher creatinine level. Since ESR level is proportional to the intensity of inflammation, it is estimated that more severe inflammatory immune response occurs in KDSS.

**Fig. 4:**
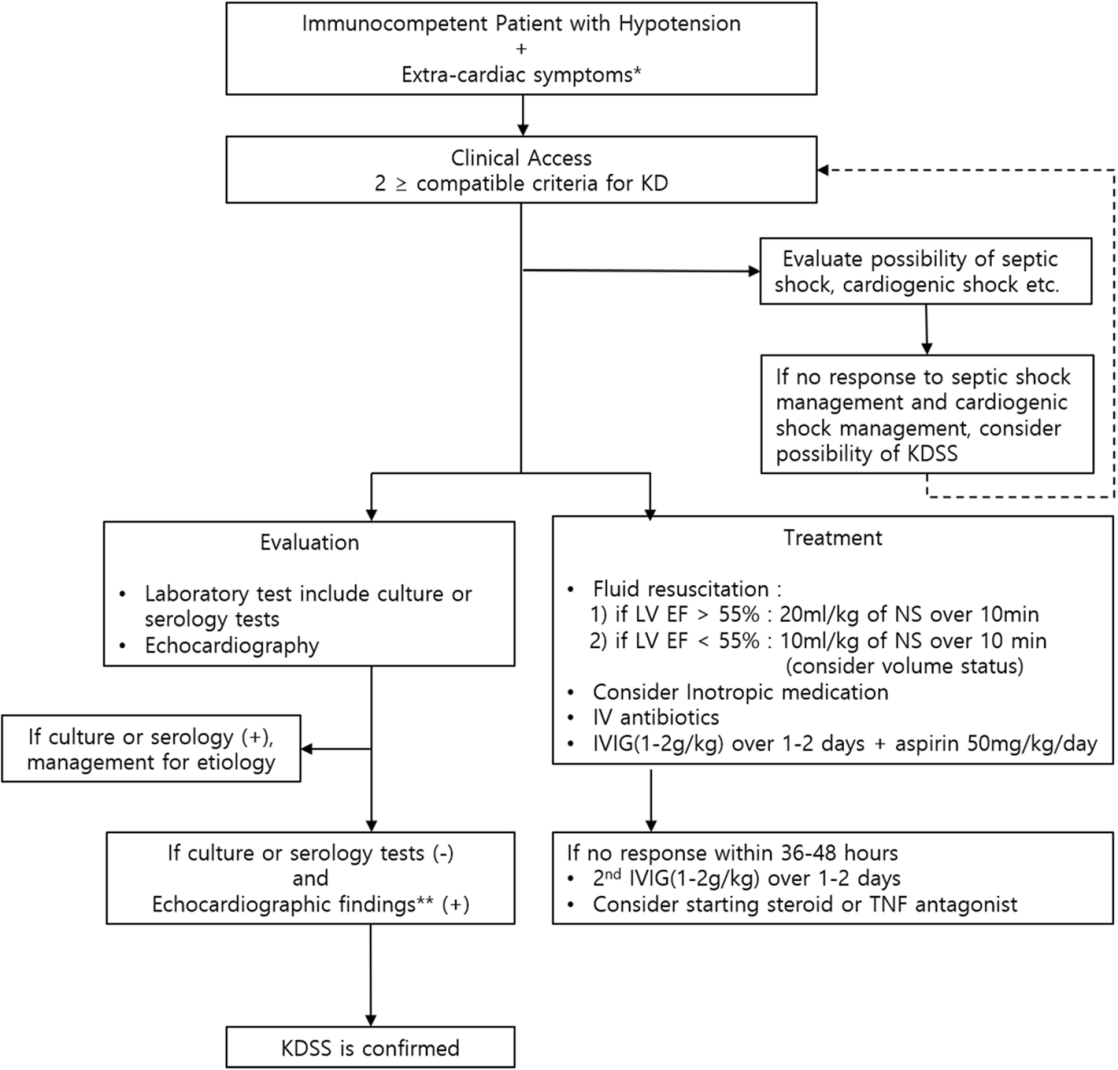
Diagnosis and treatment guideline suggested for Kawasaki disease shock syndrome. *Extra-cardiac symptoms were included gastrointestinal, respiratory, neurologic symptoms and systemic pain. ^**^Coronary artery abnormality was defined from AHA scientific statement (7). IVIG = Intravenous immunoglobulin, NS = Normal saline, TNF = Tumor necrosis factor

According to our comparison among each groups, specific symptoms and laboratory findings of each group are summarized in Supplement Table and Figure. Based on our clinical experiences, KDSS and SS group had many common findings. However, the KDSS group had Kawasaki symptoms, coronary dilation, and left ventricle dysfunction. These findings can help us differentiate KDSS from SS group. Based on our results, we suggested a differential diagnosis and treatment guideline for KDSS (Fig. 4).

### Study limitations

This study has several limitations. Our study was retrospective in nature and our case number was too small to analyze independent risk factors. This was a case-control study. KD patients were selected by seasonal matching to each case patient based on the date of admission within two weeks before and after. In addition, some patients were diagnosed by clinical manifestations. Thus, there could be a possibility of patient-selection bias.

## Conclusions

KDSS should be considered for patients with fever, Kawasaki symptoms, and hypotension. Diagnosis and treatment for patients with KDSS may be more complicated because atypical type, gastrointestinal, respiratory, and neurological symptoms, and treatment resistant type are more common. Clinical symptoms, laboratory finding, echocardiography, and serology or culture studies can be used to differentiate KDSS, SS and TSS. This study also suggested a guideline for diagnosis and treatment of KDSS.

## Supplementary Information

**Additional file 1: Supplement Table 1**. Centers for Disease control and prevention TSS Diagnostic Criteria. **Supplement Table 2**. Demographics and clinical characteristics of patients with Kawasaki disease shock syndrome, Kawasaki disease, Septic shock and Toxic shock syndrome. (continuous variables were described as median and range). **Supplement table 3**. Laboratory and Echocardiographic characteristics of patients with, Kawasaki disease shock syndrome, Kawasaki disease, Septic shock and Toxic shock syndrome. **Supplement Table 4**. Symptoms, diagnosis, and treatment of, Kawasaki disease shock syndrome, Kawasaki disease, Septic shock and Toxic shock syndrome.

**Additional file 2: Supplement Figure**. Specific symptoms and laboratory findings of each group. AKI = Acute kidney injury, CAL = Coronary artery lesion, ESR = Erythrocyte sediment rate, LV = Left ventricle, GI = Gastrointestinal, GOT = Glutamic oxalacetic transaminase, Cr = Creatinine, BUN = Blood Urea Nitrogen, HD=Hospital day, ICU=Intensive care unit, KD = Kawasaki disease, KDSS = Kawasaki disease shock syndrome, SS = Septic shock and toxic shock syndrome.

## Data Availability

The datasets used and/or analyzed during the current study are available from the corresponding author on reasonable request.

## Abbreviations

AHA: American Heart Association
AST: Aspartate aminotransferase
ALT: Alanine aminotransaminase
BUN: Blood urea nitrogen
CRP: C-reactive protein
ESR: Erythrocyte sedimentation rate
GGT: γ-glutamyl transpeptidase
IVIG: Intravenous immunoglobulin
ICU: Intensive care unit
KD: Kawasaki disease
KDSS: Kawasaki disease shock syndrome
MIS-C: Multi-system inflammatory syndrome in children
SS group: Septic shock or toxic shock syndrome
TSS: Toxic shock syndrome
