# Supplementary material for: Clinical aspects for differential diagnosis of Kawasaki disease shock syndrome: a case control study
Source: BMC Pediatr. 2021 Jan 8;21:25. doi: 10.1186/s12887-020-02488-w (PMC7790725; doi:10.1186/s12887-020-02488-w)
Supplement: Supplementary file 1 — Additional file 1: Supplement Table 1. Centers for Disease control and prevention TSS Diagnostic Criteria. Supplement Table 2. Demographics and clinical characteristics of patients with Kawasaki disease shock syndrome, Kawasaki disease, Septic shock and Toxic shock syndrome. (continuous variables were described as median and range). Supplement table 3. Laboratory and Echocardiographic characteristics of patients with, Kawasaki disease shock syndrome, Kawasaki disease, Septic shock and Toxic shock syndrome. Supplement Table 4. Symptoms, diagnosis, and treatment of, Kawasaki disease shock syndrome, Kawasaki disease, Septic shock and Toxic shock syndrome. [file 12887_2020_2488_MOESM1_ESM.docx]

Supplement Table 1. Centers for Disease control and prevention TSS Diagnostic Criteria

| TSS type | Criteria | | Classification |
| --- | --- | --- | --- |
|  | Clinical Criteria | Laboratory criteria |  |
| Staphylococcal TSS | - Fever: > 38.9C or 102.0F  - Rash with diffuse macular erythroderma  - Desquamation 1–2 weeks after rash onset  - Hypotension with SBP≤90mmHg (adults)  or ≤5^th^ percentile by age (<16 years old)  - Multi-organ involvement (three or more systems): Gastrointestinal (vomiting/diarrhea),  Muscular (severe myalgias or creatine kinase≥ 2 times upper limit of normal),  Mucous membrane involvement,  Renal (BUN or Cr≥2 times upper limit of normal or urinary sediment with pyuria with no urinary tract infection),  Hepatic (total bilirubin, ALT or AST ≥ 2 times upper limit of normal)  Hematologic (platelets ≤ 100,000/mm3),  Neurologic (alteration in consciousness without focal neurologic signs when fever and hypotension are absent | (These must be negative if obtained)  -Blood or cerebrospinal fluid cultures (blood cultures may be positive for Staphylococcus aureus)  - Serologies for Rocky Mountain spotted fever, leptospirosis, or measles | - Probable  ˃4 clinical criteria and laboratory criteria met  - Confirmed  5 clinical criteria and laboratory criteria met, including desquamation |
| Streptococcal TSS | - Hypotension with SBP≤90 mm Hg (adults) or ≤5^th^ percentile by age (<16 years old)  - Multi-organ involvement (two or more systems): Gastrointestinal(vomiting/diarrhea),  Muscular (severe myalgias or creatine kinase ≥2 times upper limit of normal),  Mucous membrane involvement,  Renal (Cr≥2 mL/dL or Cr > 2 times upper limit of normal, > twofold elevation from patient baseline), Hepatic (total bilirubin, ALT, AST≥2 times upper limit of normal),  Hematologic (platelets≤100,000/mm3, disseminated intravascular coagulation, or > twofold elevation from patient baseline),  Acute respiratory distress syndrome,  Skin (generalized erythematous macular rash that can desquamate),  Soft tissue necrosis (gangrene, myositis, necrotizing fasciitis) | - Group A streptococcus isolation from culture | - Probable  All clinical criteria met and absence of other etiology for illness with isolation of group A Streptococcus from nonsterile site.  - Confirmed  All clinical criteria met and isolation of group A Streptococcus from sterile site (blood, cerebrospinal fluid, synovial fluid, pleural/pericardial fluid) |

TSS = toxic shock syndrome; SBP = systolic blood pressure; BUN = blood urea nitrogen; Cr = creatinine; ALT = alanine aminotransferase; AST = aspartate aminotransferase.

Supplement Table 2. Demographics and clinical characteristics of patients with Kawasaki disease shock syndrome, Kawasaki disease, Septic shock and Toxic shock syndrome. (continuous variables were described as median and range)

|  | KDSS (n=13) | KD (n=91) | SS (n=16) | TSS (n=13) |
| --- | --- | --- | --- | --- |
| Age, (years) | 5.1 (0.5-10.6) | 2.3 (0.2-8.4) | 5.9 (0.3-15.9) | 9.3(1.2-19.9) |
| Male (%) | 46.2% (6/13) | 58.2% (53/91) | 62.5% (10/16) | 69.2% (9/13) |
| Fever duration (days) | 11 (8-23) | 8 (3-29) | 7.5(1-114) | 7(5-33) |
| Total hospital day (days) | 18 (6-31) | 6 (3-18) | 14(7-225) | 13(7-74) |
| Follow up duration (months) | 10 (1-105) | 13 (0-175) | 1(0-105) | 4(0-111) |
| ICU care (%) | 53.8 % (7/13) | 0.0% (0/91) | 68.8%(11/16) | 53.8%(7/13) |
| ICU care duration (days) | 4 (0 - 11) | 0 | 4(0-11) | 3(0-20) |
| Inotropic drugs (%) | 92.3 % (12/13)^§^ | 1.1% (1/91) | 100%(16/16) | 69.2%(9/13) |
| Respiratory support |  |  |  |  |
| - No support | 30.8 % (4/13) | 100% (91/91) | 37.5% (6/16) | 61.5% (8/13) |
| - Oxygen delivery | 53.8% (7/13) | 0.0% (0/91) | 12.5% (2/16) | 15.4% (2/13) |
| - Mechanical ventilation | 15.4% (2/13) | 0.0% (0/91) | 50.0% (8/16) | 23.1% (3/13) |
| Mortality (%) | 0.0 % (0/13) | 0.0% (0/91) | 18.8% (3/16) | 0.0% (0/13) |

KDSS = Kawasaki disease shock syndrome, KD = Kawasaki disease, SS = Septic shock, TSS=toxic shock syndrome, ICU = Intensive care unit

Supplement table 3. Laboratory and Echocardiographic characteristics of patients with, Kawasaki disease shock syndrome, Kawasaki disease, Septic shock and Toxic shock syndrome.

|  | KDSS(n=13) | KD(n=91) | SS (n=16) | TSS (n=13) |
| --- | --- | --- | --- | --- |
| Laboratory findings |  |  |  |  |
| WBC (10^3^/㎕) | 11.7 (6.8-25.5) | 12.6 (3.5-24.8) | 11.4 (3.6-24.7) | 12.1 (4.1-34.7) |
| Lowest hemoglobin (g/dL) | 9.1 (5.5-11.3) | 10.7 (6.9-13.0) | 8.5 (4.9-13.3) | 10.0 (8.1-11.5) |
| Lowest Platelet (10^3^/㎕) | 125 (23.0-299.0) | 311 (41.0-782.0) | 99.0 (5.0-221.0) | 107.0 (12.0-260.0) |
| Highest CRP(mg/dL) | 21.3 (10.4-32.0) | 6.8 (1.0-26.4) | 18.2 (3.2-35.1) | 15.1 (3.6-35.9) |
| Highest ESR(mm/hr) | 75.0 (25-108) | 72.0 (5-120) | 52.0 (2-120) | 35.0 (6-44) |
| Highest GOT (IU/L) | 40.0 (18-152) | 38.5 (21-1687) | 59.5 (18-8900) | 83.0 (27-1370) |
| Highest GPT (IU/L) | 57.0 (12-249) | 38.0 (7-1250) | 61.5 (10-2290) | 59.0 (15-1253) |
| Highest Total bilirubin(mg/dL) | 1.3 (0.3-4.9) | 0.6 (0.2-13.0) | 1.3 (0.5-53.9) | 2.2 (0.5-21.7) |
| Highest Cr (mg/dL) | 0.6 (0.4-1.9) | 0.4 (0.2-0.8) | 0.7 (0.39-6.9) | 1.0 (0.37-4.8) |
| Highest BUN (mg/dL) | 18.0 (6.0-53.0) | 10.0 (3.0-18.0) | 16.0 (9.0-267.0) | 34.0 (9.0-100.0) |
| Lowest albumin (g/dL) | 2.5 (2.1-2.9) | 3.3 (2.3-4.5) | 2.6 (1.5-3.6) | 2.5 (2.0-3.3) |
| Lowest sodium (mmol/L) | 130.0 (115.0-140.0) | 135.0 (129-140) | 133.5 (125.0-139.0) | 132.0 (126.0-137.0) |
| Echocardiographic findings |  |  |  |  |
| Intracardiac anomaly | 0.0% (0/13) | 7.7% (7/91) | 0.0% (0/9) | 37.5% (3/8) |
| Coronary artery aneurysm | 23.1% (3/13) | 4.4% (4/91) | 0.0% (0/4) | 0.0%(0/8) |
| EF < 55% | 46.2% (6/13) | 4.5% (4/88) | 12.5% (1/8) | 12.5% (1/8) |
| Lowest EF | 56.9 (49.0-71.6) | 62.3 (45.6-74.8) | 64.8 (49.0-73.0) | 60.5 (53.9-74.3) |

Data were described as median and range.

*WBC= White blood cell, CRP = C-reactive protein, ESR = Erythrocyte Sedimentation Rate, GOT = Glutamic oxalacetic transaminase, GPT = glutamate-pyruvate transaminase, Cr = creatinine, BUN = Blood Urea Nitrogen, EF = Ejection fraction, KD = Kawasaki disease, KDSS = Kawasaki disease shock syndrome, SS = Septic shock, TSS= Toxic shock syndrome

Supplement Table 4. Symptoms, diagnosis, and treatment of, Kawasaki disease shock syndrome, Kawasaki disease, Septic shock and Toxic shock syndrome

|  | KDSS(n=13) | KD(n=91) | SS (n=16) | TSS (n=13) |
| --- | --- | --- | --- | --- |
| Kawasaki features |  |  |  |  |
| Conjunctival injection | 92.3% (12/13) | 91.2% (83/91) | 18.8% (3/16) | 0.0% (0/13) |
| Oropharyngeal changes | 84.6% (11/13) | 81.3% (74/91) | 6.3% (1/16) | 30.8% (4/13) |
| Polymorphous rash | 76.9% (10/13) | 84.6% (77/91) | 43.8% (7/16) | 100.0% (13/13) |
| Cervical lymphadenopathy | 69.2% (9/13) | 44.0% (40/91) | 6.3% (1/16) | 0.0% (0/13) |
| Extremity changes | 92.3% (12/13) | 67.0% (61/91) | 12.5% (2/16) | 69.2% (9/13) |
| Associated symptoms |  |  |  |  |
| Gastrointestinal symptoms | 84.6% (11/13) | 26.4% (24/91) | 75.0% (12/16) | 92.3% (12/13) |
| Respiratory symptoms | 76.9% (10/13) | 9.9% (9/91) | 56.3% (9/16) | 23.1% (3/13) |
| Neurologic symptoms | 15.4% (2/13) | 2.2% (2/91) | 37.5% (6/16) | 61.5% (8/13) |
| Pantalgia | 53.8% (7/13) | 3.3% (3/91) | 25.0% (4/16) | 53.8% (7/13) |
| Pleural effusion | 76.9% (10/13) | 2.2% (2/91) | 43.8% (7/16) | 38.5% (5/13) |
| Organ damage | 92.3% (12/13) | 20.9%(19/91) | 75.0% (12/16) | 92.3% (12/13) |
| Diagnosis |  |  |  |  |
| Initial diagnosis of KD | 23.1% (3/13) | 80.2% (73/91) | 0.0% (0/16) | 0.0% (0/13) |
| Complete KD | 46.2% (6/13) | 67.0% (61/91) | 0.0% (0/16) | 0.0% (0/13) |
| Incomplete KD | 38.5% (5/13) | 29.7% (27/91) | 0.0% (0/16) | 0.0% (0/13) |
| Treatment |  |  |  |  |
| 1^st^ IVIG | 84.6% (11/13) | 98.9% (90/91) | 50.0% (8/16) | 53.8% (7/13) |
| 2^nd^ IVIG | 76.9% (10/13) | 19.8% (18/91) | 25.0% (4/16) | 7.7% (1/13) |
| Oral prednisolone | 7.7% (1/13) | 11.0% (10/91) | 0.0% (0/16) | 0.0% (0/13) |
| Methylprednisolone pulse therapy | 38.5% (5/13) | 11.0% (10/91) | 0.0% (0/16) | 0.0% (0/13) |
| Infliximab | 15.4% (2/13) | 0.0% (0/91) | 0.0% (0/16) | 0.0% (0/13) |
| Antibiotics | 92.3% (12/13) | 15.4% (14/91) | 100.0% (16/16) | 100.0% (13/13) |

IVIG = Intravenous immunoglobulin, KD = Kawasaki disease, KDSS = Kawasaki disease shock syndrome, SS = Septic shock, TSS=toxic shock syndrome
